# Effectiveness of home-based rehabilitation program in minimizing disability and secondary falls after a hip fracture: Protocol for a randomized controlled trial

**DOI:** 10.1016/j.isjp.2020.06.002

**Published:** 2020-06-18

**Authors:** Anum Sadruddin Pidani, Saniya Sabzwari, Khabir Ahmad, Ata Mohammed, Shahryar Noordin

**Affiliations:** a Department of Surgery, Aga Khan University, Karachi, Pakistan; b Department of Family Medicine, Aga Khan University, Karachi, Pakistan; c Department of Physiotherapy, Aga Khan University, Karachi, Pakistan

**Keywords:** THR, Total hip replacement, CTU, Clinical trial unit, ERC, Ethical Review committee, Hip fracture, Secondary falls, Disability, Rehabilitation, Physical activity, Elderly population

## Abstract

**Introduction:**

Hip fractures are a major health problem globally and are associated with increased morbidity, mortality, and substantial economic costs. Successful operative treatment of hip fracture patients is necessary for the optimization of post-op mobility and functional recovery of the patient. Rehabilitation after surgical stabilization of a hip fracture is crucial in order to restore pre-fracture function and to avoid long-term institutionalization. In particular ongoing exercise which targets balance can prevent up to 40% of falls. Therefore, we have designed a post-discharge home-based physical rehabilitation intervention program to minimize disability and falls in this high-risk elderly population.

**Methods and analysis:**

The study will be an open label, simple randomized controlled trial at a single hospital. The two arms will be equally allocated on a 1:1 ratio into intervention and control groups. The control arm will receive the usual standard postoperative rehabilitation. The intervention group will receive an extended home-based rehabilitation program twice a week continued for 3 months (12 weeks) after discharge. The Primary outcome of the study is occurrence of falls. Falls will be measured at 3, 6, 12, and 24 months by research-assistant follow-up telephone calls for both the groups. Mobility-related disability will be measured with a self-reported test at every routine follow-up for up to two years using a performance-based short battery tool. Negative binomial regression model will be used to compare number of falls in both the groups by computing incidence ratio rates.

**Ethics and dissemination:**

Approval for the conduction of this study has been taken from the Ethical Review Committee (ERC) of the institution. Evidences which will be obtained from this study will facilitate to propose changes in existing guidelines and policies for treating fall and hip fracture patients.

Trial registration

This trial is registered on clinicaltrials.gov ID: NCT04108793.

## Highlights

• Hip fractures are associated with increased morbidity, mortality, and substantial economic costs.
• A randomized controlled trial is design to compare home-based physical intervention and standard care.
• The primary outcome is the assessment of occurrence of secondary falls in elderly hip fracture patients.

## 1 Introduction

Falls and fractures in older people are of major public health significance. One in three older people experience one or more falls each year [1] and about one in six 50-year-old women will have at least one hip fracture before they die [2]. This propensity for fall-related injury in elderly persons stems from a high prevalence of comorbid diseases such as osteoporosis and age-related physiological decline (e.g. slower reflexes) that make even a relatively mild fall, potentially dangerous. With a rising elderly population, hip fracture incidence and cost are expected to grow substantially [3]. It is estimated that by 2050 the total global cost of hip fractures will be $131.5 billion annually [4]. Unfortunately, outcomes after hip fracture are often poor [5] with high mortality rates and decreased functionality and mobility in survivors [6]. Henceforth, fall is major giant of elderly people in community settings [7]. It poses major social, psychological and economic burden to individuals and their families.

In Pakistan, there is a lack of large scale epidemiological studies resulting in lack of statistical data on hip fractures. An ultrasound based study conducted throughout the country concluded that currently there are 9.91 million people suffering from osteoporosis [8]. They further added that the numbers estimated will rise up to 11.3 million by 2020 and 12.91 million by 2050. In addition, 75% of the patients suffering from osteoporosis in Pakistan have had hip fractures [9]. On the other hand, considering low capital per income, the overall cost for treating patients with hip fracture is very high eventually leading to increased healthcare burden.

Total hip replacement (THR) remains the ultimate treatment option for patients suffering from hip fractures and severe hip osteoarthritis [10]. Rehabilitation after hip fracture surgery is the most critical component of post-surgical care and has been associated with better functional outcomes [10]. According to McMeeken & Galea (2007), the key feature of THR rehabilitation program is strengthening of muscles around the affected joint and improvement in gait and joint stability. A systematic review of 88 trials conducted in 2016 reports significant effect of home-based exercise program post-surgery on strengthening of leg muscles and overall functionality [11]. However, adherence to exercise programs is a major issue identified in both public health researches and clinical practice. There is an evidence that long term adherence to exercise may be better with home-based rehabilitation programs rather than institution-based programs especially for older adults [12].

The development and implementation of effective rehabilitation strategies to minimize disability and secondary falls predominantly among older people is an urgent public health challenge due to the increasing proportion of older people in the global world [13], [14], [15]. Around 962 million people worldwide are aged 60 years and older [16] and this number is predicted to increase to 2.1 billion by 2050 which will represent one eighth of the global population [17]. Developing countries are predicted to have a 140 percent increase in older people by 2030 [18]. A study conducted by students and faculties at nurse-managed clinic in US concluded that recurrent falls are a common reason for admission of previously independent elderly persons to long-term care institutions [19]. Bezon et al. [20] conducted a study in British community reported falls to be a major reason for 40% of nursing home admissions. Fear of falling and the post-fall anxiety syndrome are also well recognized as negative consequences of falls. The loss of self-confidence to ambulate safely can result in self-imposed functional limitations [21], [22], [23].

For many individuals, the risk of falls and disability after hospital discharge decreases with time. However, for a significant proportion, this is not the case [24]. Many patients after discharge are readmitted with second fracture to the hospitals due to lack of acute rehabilitation programs [25]. Therefore, we have designed a post-discharge home-based physical rehabilitation intervention program to minimize disability and falls in this high-risk elderly population. This study will evaluate the effectiveness of 12 week home-based physiotherapy program in reduction of secondary falls and improvement in physical mobility of elderly patients after hip fracture surgery.

## 2 Methods

### 2.1 Study design

This study will be an open label, simple randomized controlled trial at a single hospital. The two arms will be equally allocated on a 1:1 ratio into intervention and control groups. The control arm will receive the usual standard postoperative rehabilitation after a bipolar hemiarthroplasty/total hip arthroplasty which will include in hospital rehabilitation and a maximum of 5 physiotherapist visits postoperatively, arranged and funded by the patient as feasible. The intervention group will receive an extended home-based rehabilitation program twice a week continued for 3 months (12 weeks) after discharge funded by the study.

### 2.2 Recruitment

The study will be conducted solely at Aga Khan University Hospital. The care providers involved with the study will include Orthopaedic consultants, Family medicine physician, physiotherapist, and Orthopaedic nurses. We will recruit 224 elderly patients aged 60 years and above undergoing hip fracture surgery who will be randomly divided into intervention and control arms. Participants will be enrolled by research-assistant over a 19‐month period from Emergency Room and Orthopedic outpatient clinics. This study will be conducted over a period of 24 months with ongoing recruitment and follow-ups after intervention.

#### 2.2.1 Inclusion criteria

To be included in the study patients must meet the following inclusion criteria: 1) age ≥ 60 years; 2) able to walk independently with or without a walking frame prior to the fracture; 3) diagnosis of proximal femoral fracture; 4) history of fall; 5) surgical procedure Bipolar hemiarthroplasty / total hip replacement and postoperative ambulatory status weight bearing as tolerated.

#### 2.2.2 Exclusion criteria

Patients will be ineligible to participate in the study if they are unable to walk more than one meter despite assistance with a walking aid and/or another person, are legally blind, have a progressive neurological disease (e.g. Parkinson’s disease, dementia) or low Conscious level (low GCS) or suffer from any medical condition precluding exercise (e.g. unstable cardiac disease) or other uncontrolled chronic conditions that would interfere with the safety and conduct of the training and testing protocol or interpretation of results. Moreover, Patients undergoing dynamic hip screw (DHS) will be excluded from the study due the different postoperative weight bearing regimens including non-weight bearing, toe touch, partial and full weight-bearing. Most of the patients with a bipolar hemiarthroplasty/ total hip arthroplasty are allowed full weight bearing postoperative and this will therefore keep the groups homogenous. We will utilize medical record files to assess eligibility criteria of patients for participation in the study.

### 2.3 Home-based rehabilitation program

Home-base rehabilitation program is an extended 12 weeks home-based strength retraining program of progressive balance and lower limb strengthening exercises twice a week for 3 months as well as evidence based cueing strategies to reduce freezing of gait. All exercises will include 5 minutes warm-up exercises. The lower limb extensor muscle groups, which act to prevent collapse of the lower limb (hip & knee extensors and ankle plantar flexors) will be targeted with exercises designed to enhance postural control (i.e. balance) and muscle strength. The balance exercises include standing with a decreased base of support, forwards and sideways stepping/walking, and graded reaching activities in standing. Strengthening exercises will include sit-to-stand, forward and lateral step-ups onto a small block, semi squats and heel raises in standing. Standard principles governing frequency, volume, duration, intensity and progression of exercise will be applied. Cueing strategies will be used to reduce freezing. These will include identifying appropriate methods of cueing (cognitive, auditory, and somatosensory) for each participant and incorporating these cues into everyday activities.

The physiotherapists will deliver home-based rehabilitation program with sufficient clinical experience with older population. For each patient randomized to the intervention group, a physiotherapist will visit the patient’s home and will prescribe a selection of exercises at the first visit. The same physiotherapist will return twice a week for 3 months to make progressive adjustments to the exercise protocol according to the home-base rehabilitation program exercise manual.

### 2.4 Data collection

**Fig. 1:**
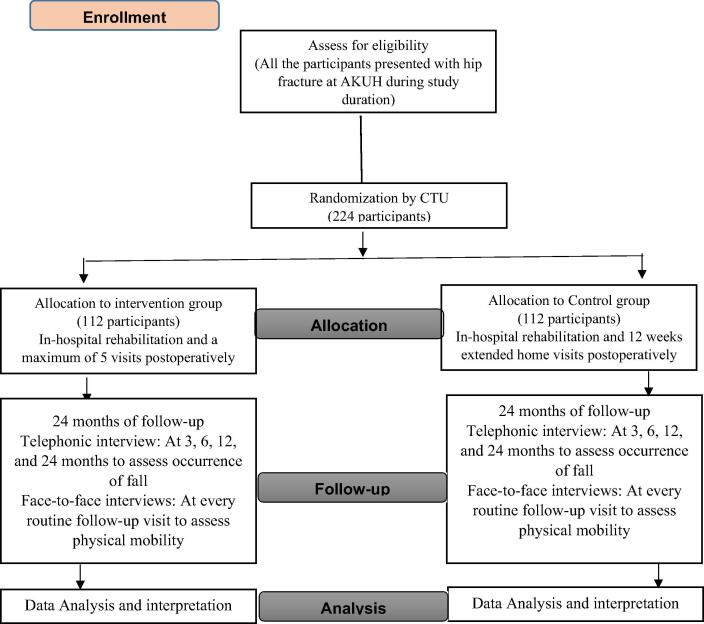
Study design flow-chart.

Participant will be enrolled into the study after the informed consent process has been completed, and the patients have met eligibility criteria. Patient consent for study participation will be obtained during the hospital stay by the research-assistant. Also, medical clearance will be required from each patient’s primary surgeon to certify him / her as able to participate in moderate-intensity rehabilitation program before taking part in the study. A research-assistant will perform a baseline assessment using a pre-structured questionnaire to assess baseline characteristics including age, gender, marital status, educational status, socio-economic, BMI, past medical history, medication, assessment of neurological status (Glasgow coma scale (GCS) will be used to assess conscious level of patient) functional status, use of ambulatory aids, number of previous falls, Type of fracture, and surgical repair type. A physiotherapist will perform assessment of mobility-related disabilities using performance-based mobility measure at baseline. The scores at baseline will be recorded for all the patients (intervention and control arms). Lastly, fall risk assessment, geriatric assessment, and general fall prevention teaching will be done by geriatric expert physician at the time of enrolment for both the groups. All the measurements will be done before hip fracture surgery (preoperative assessment). All the adverse events (defined as a significant injury or medical event that causes the participant to seek attention from a health professional or limit their activities) will be monitored and recorded throughout the study during the follow-up telephone calls to each participant. All the adverse events will be covered through institutional insurance coverage. Fig. 1 shows the details of participant’s recruitment, randomization, and follow-up.

### 2.5 Outcomes assessment

#### 2.5.1 Primary outcome

The Primary outcome of the study is occurrence of falls. Falls will be measured at 3, 6, 12, and 24 months by research-assistant follow-up telephone calls for both the groups. A structured pre-tested questionnaire will be used to assess falls on telephonic interview. The interview will take 5–10 minutes of each participants to assess occurrence of any new fall incident after discharge. Falls will be assessed by comparing the number of falls in intervention and control groups.

#### 2.5.2 Secondary outcome

Physical Mobility and Mobility-related disability will be measured with a self-reported test at every routine follow-up for up to two years using a performance-based test as per Haley et al. [26]. The performance-based mobility measure will be the lower extremity Summary Performance Score version of the Short Physical Performance Battery (SPPB) [27]. This battery gives a composite score based on timed performance of three mobility tasks: the ability to stand up for up to 10 seconds with feet in different positions (together side by side, semi-tandem and tandem), 4-meter walk and time to rise from a chair 5 times. The short performance based mobility tool is a valid and reliable tool with test retest validity ranging from 0.83 to 0.89. Study conducted by Gómez and colleagues in 2013 on adults between 65 to 74 years of age residing in the Andes Mountains of Colombia reported Test-retest reliability of 0.87 of the SPPB in Spanish version [28]. Another study conducted in Quebec and Brazil by Freire and colleagues in 2012 reported the validity and reliability of SPPB in diverse populations ranging from 0.83 to 0.89 [29]. This short performance battery tool had been validated in varied languages in different regions but not in Urdu language. Therefore, to use this tool in the study, we will perform back and forward translation of SPPB in Urdu language and will send it to 5–7 clinical experts to rate it for clearance and relevance in order to calculate the validity of tool in Urdu language.

### 2.6 Follow-ups

For follow-ups, telephone calls and face to face interviews will be utilized. All the Patients (both groups) will be contacted after 3 months, 6 months, 12 months, and 24 months (Fig. 1) for the period of two years via telephone call to assess occurrence of fall. The call will take approximately 5–10 minutes of each patient. While, the performance-based mobility measure will be marked at every routine follow-up for up to two years using face to face interview to assess the overall trend. Furthermore, anthropometric assessment will also be done to assess weight loss at each follow-up.

### 2.7 Sample size

Open Epi was [30] used to compute sample size for the study. The retrospective analyses of studies have showed that reported incidence of falls in elderly patients is between 30–40% globally [31]. Therefore, 40 % fall rates were considered for control group to calculate sample size. Moreover, based on evidences from previous studies, home-based rehabilitation program can reduce incidence of falls up to 20% [11]. Henceforth, considering 40% fall rates in control group with an absolute risk reduction of 20% in intervention group, power of 0.8 and two sided confidence interval of 95% yields a sample size of 94 per group. Adding 20% attrition rate, the final sample size is calculated to be 112 per group.

### 2.8 Randomization

Randomization will be performed by Clinical Trial Unit (CTU) at Aga Khan University using a computerized random-number generator. Randomization will be restricted by a permuted block design of size four or six with stratification to one of two study arms to ensure that both the study group receives half of the participants. CTU will sealed the randomization codes in sequentially numbered opaque envelopes which will be assigned to participants in their order of recruitment by the research officer at CTU not involved in the study.

### 2.9 Blinding

This study is an open label trial where patients, orthopedic surgeons, Nurses, and physical therapist will be aware of the intervention group. Therefore, no blinding or allocation concealment will be performed. However, the research assistant who will assess occurrence of fall on telephone call will be kept blinded to ensure blinded data assessing.

### 2.10 Statistical analysis

Intention to treat Analysis will be performed for both the groups. All the analysis will be done on STATA version 15.0 [32]. Baseline characteristics for both groups and changes from baseline to 6 months and baseline to 2 year will be compared in the two groups using the χ2 test, Mann-Whitney, or Student's t-test as applicable. The number of falls in both the groups will be compared by computing incidence ratio rates using negative binomial regression models after adjusting for baseline differences in fall rates along with incomplete/loss to follow‐up in a small percentage of subjects. Between group comparisons of final test performance for the continuously-scored outcome measures will be made using General Linear Models (ANCOVA) controlled for pre-test performance.

### 2.11 Data management

Every questionnaire, screening tool, and consent will contain a printed study ID unique for each participant. All the data entry and analysis will be done using the study ID. Hard copies of the data will be kept in securely locked filling cabinets at department of surgery and only study principle investigator and Co-I will have access to the hard copies. Moreover, Password protected computer and data bases will be used to maintain confidentiality of electronic data. In addition, the backup files for electronic data will also be maintained to prevent loss of data.

### 2.12 Ethics and dissemination

Approval for the conduction of this study has been taken from the Ethical Review Committee (ERC) of the institution. Informed written consent to participate will be obtained from all participants recruited in the study. The findings of this study will be communicated with all the healthcare providers including physicians, surgeons, and nurses via Seminar, symposium, scientific paper, and presentation. Besides, we will utilize news brief and brochures to communicate findings with local communities and study participants.

## 3 International Journal of Surgery Protocols

The following information is required for submission. Please note that failure to respond to these questions/statements will mean your submission will be returned. If you have nothing to declare in any of these categories then this should be stated.

## 4 Please state any conflicts of interest

All authors must disclose any financial and personal relationships with other people or organisations that could inappropriately influence (bias) their work. Examples of potential conflicts of interest include employment, consultancies, stock ownership, honoraria, paid expert testimony, patent applications/registrations, and grants or other funding.

## 5 Please state any sources of funding for your research

All sources of funding should be declared as an acknowledgement at the end of the text. Authors should declare the role of study sponsors, if any, in the collection, analysis and interpretation of data; in the writing of the manuscript; and in the decision to submit the manuscript for publication. If the study sponsors had no such involvement, the authors should so state.

## Ethical Approval

Research studies involving patients require ethical approval. Please state whether approval has been given, name the relevant ethics committee and the state the reference number for their judgement.

## Author contribution

Please specify the contribution of each author to the paper, e.g. study concept or design, data collection, data analysis or interpretation, writing the paper, others, who have contributed in other ways, should be listed as contributors.

## 8 Guarantor

The Guarantor is the one or more people who accept full responsibility for the work and/or the conduct of the study, had access to the data, and controlled the decision to publish
